# Impairments of the ipsilesional upper-extremity in the first 6-months post-stroke

**DOI:** 10.1186/s12984-023-01230-8

**Published:** 2023-08-14

**Authors:** Donovan B. Smith, Stephen H. Scott, Jennifer A. Semrau, Sean P. Dukelow

**Affiliations:** 1 Department of Clinical Neurosciences, Hotchkiss Brain Institute, University of Calgary, 1403 29th Street NW, Foothills Medical Centre, South Tower, Room 905, Calgary, AB T2N 2T9 Canada; 2 Department of Biomedical and Molecular Sciences, Queen’s University, Kingston, ON Canada; 3 Department of Kinesiology and Applied Physiology, University of Delaware, Newark, Delaware, USA

**Keywords:** Stroke, Rehabilitation, Ipsilesional, Robotics, Reaching, Motor impairment

## Abstract

**Background:**

Ipsilesional motor impairments of the arm are common after stroke. Previous studies have suggested that severity of contralesional arm impairment and/or hemisphere of lesion may predict the severity of ipsilesional arm impairments. Historically, these impairments have been assessed using clinical scales, which are less sensitive than robot-based measures of sensorimotor performance. Therefore, the objective of this study was to characterize progression of ipsilesional arm motor impairments using a robot-based assessment of motor function over the first 6-months post-stroke and quantify their relationship to (1) contralesional arm impairment severity and (2) stroke-lesioned hemisphere.

**Methods:**

A total of 106 participants with first-time, unilateral stroke completed a unilateral assessment of arm motor impairment (visually guided reaching task) using the Kinarm Exoskeleton. Participants completed the assessment along with a battery of clinical measures with both ipsilesional and contralesional arms at 1-, 6-, 12-, and 26-weeks post-stroke.

**Results:**

Robotic assessment of arm motor function revealed a higher incidence of ipsilesional arm impairment than clinical measures immediately post-stroke. The incidence of ipsilesional arm impairments decreased from 47 to 14% across the study period. Kolmogorov–Smirnov tests revealed that ipsilesional arm impairment severity, as measured by our task, was not related to which hemisphere was lesioned. The severity of ipsilesional arm impairments was variable but displayed moderate significant relationships to contralesional arm impairment severity with some robot-based parameters.

**Conclusions:**

Ipsilesional arm impairments were variable. They displayed relationships of varying strength with contralesional impairments and were not well predicted by lesioned hemisphere. With standard clinical care, 86% of ipsilesional impairments recovered by 6-months post-stroke.

**Supplementary Information:**

The online version contains supplementary material available at 10.1186/s12984-023-01230-8.

## Introduction

Stroke is one of the leading causes of death and disability around the world, with over 13.7 million new cases each year [1]. Upper extremity motor impairment after stroke can be profound, impacting nearly 75% of survivors [2]. These impairments have traditionally been viewed as mainly affecting the contralesional limb, and as a result, recovery has primarily been gauged by contralesional arm motor performance [3–5]. Previous work has stressed the importance of analyzing ipsilesional impairments throughout stroke recovery [6, 7], and has demonstrated that up to 37% of stroke survivors experience motor impairments in their ipsilesional limb [8]. In cases where the contralesional limb is unable to recover sufficiently, the ipsilesional limb then becomes the dominant limb required to complete activities of daily living [9]. If the ipsilesional limb is also impaired, activities of daily living can become increasingly difficult to complete [10], and functional recovery could prove challenging [11].

A number of studies have examined ipsilesional arm impairments after stroke [8, 11–14]. While ipsilesional arm impairments seem to improve with time post-stroke [6, 15, 16], there are incongruent findings around the influence of the stroke-lesioned hemisphere on ipsilesional impairments. Some studies have suggested that left-hemisphere damage impacts movement trajectory direction and curvature, and right-hemisphere damage impacts movement endpoint control [14, 17–19]. As these studies only recruited right-hand dominant chronic stroke participants, the findings must be interpreted carefully, particularly in those with subacute stroke. Other studies have suggested no hemispheric differences are present in ipsilesional arm motor behaviour following stroke [6, 8, 20, 21]. This incongruency must be sorted out to provide a definitive answer as to if lesioned hemisphere impacts the severity and/or type of motor impairments observed in the ipsilesional arm, better informing clinical practice when prescribing rehabilitation for stroke survivors [18].

Another area of debate in the literature focuses on whether ipsilesional motor impairments scale with the severity of contralesional motor impairments. The current literature presents conflicting results, with a recent study finding that ipsilesional impairments scale with the severity of the contralesional impairments [18], yet another recent study suggesting that ipsilesional impairments are unrelated to contralesional impairment [6]. However, one of these studies examined behaviour in a smaller subacute stroke sample (n = 19) [6], and the other focused on chronic stroke [18]. Better understanding the relationship between the severity of ipsilesional and contralesional arm impairments is important, and a larger, longitudinal study is required to determine how this relationship changes.

The main goal of the present study was to characterize ipsilesional arm motor impairments throughout the first 6-months post-stroke. Here, we examined how motor impairments change from their initial presentation in the subacute phase at 1-week post stroke to the chronic phase at 6-months post-stroke in both the ipsilesional and contralesional arm. Second, we determined the proportion of participants with persistent ipsilesional and contralesional motor impairments at 6-months post-stroke. Third, we determined if ipsilesional arm motor impairments scaled with the severity of contralesional arm motor impairments. Last, we determined if the side of the stroke-lesioned hemisphere was related to the severity and type of motor impairments seen in the ipsilesional arm.

## Methods

We recruited inpatient participants with stroke from two locations in Calgary, Alberta (Foothills Hospital and the Dr. Vernon Fanning Centre). Inclusion criteria were: first-time unilateral stroke, age ≥ 18, and the ability to follow task instructions. Exclusion criteria were: bilateral stroke (as confirmed by neuroimaging), brainstem and/or cerebellar stroke, other neurological disease or injury (e.g. Multiple Sclerosis), orthopedic injuries to the upper extremities, or the presence of apraxia [10, 22]. These criteria allowed participants with a range of motor impairments to participate in the study and ensured that individuals who might have more subtle impairments not easily detected with traditional clinical assessments were included. Participants were assessed using both clinical and robotic measures of post-stroke impairment at 4 separate time points; 1-, 6-, 12-, and 26-weeks post-stroke. The study was approved by the University of Calgary Research Ethics Board and all participants provided informed consent.

### Robotic assessments of upper extremity impairment

**Fig. 1:**
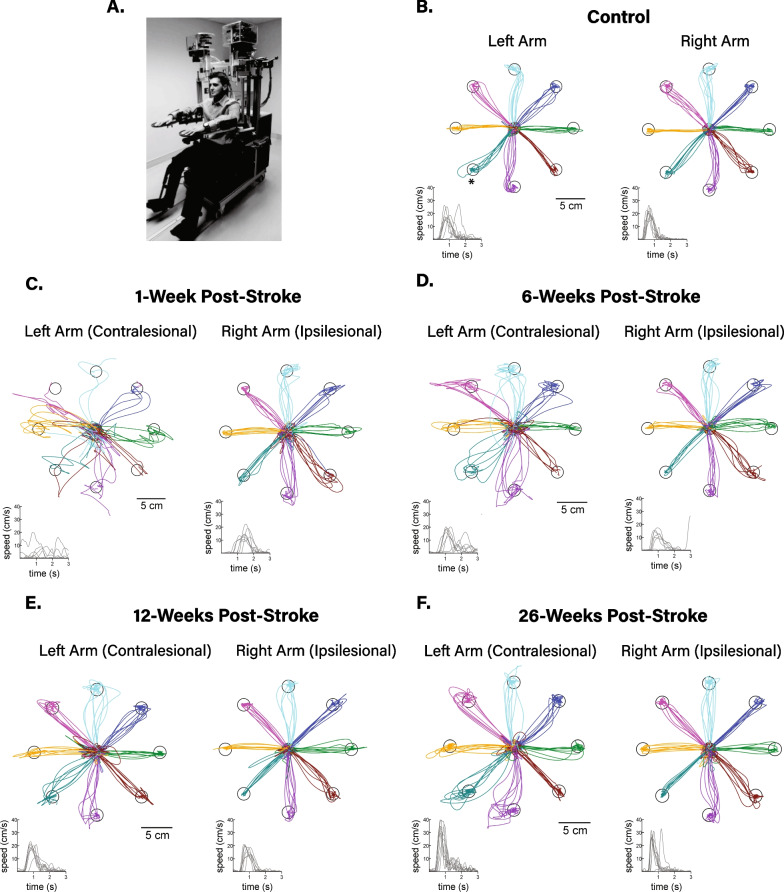
Exemplar reaching movements on the visually guided reaching task. **A** Picture of the Kinarm robotic exoskeleton. **B** Exemplar position and hand speed data from a healthy control subject. **C**–**F** Exemplar position and hand speed data from a participant with stroke at 1 (**C**), 6 (**D**), 12 (**E**), and 26 weeks **F** post-stroke. Asterisk in **B** denotes the target direction corresponding to the hand speed traces in panels **B** through **F**

To assess upper extremity impairment, participants performed a visually guided reaching (VGR) task (Fig. 1) using the Kinarm Exoskeleton Lab (Kinarm, Kingston, Ontario, Canada, Fig. 1A). Participants were seated in the wheelchair base of the robotic exoskeleton with their arms supported against gravity by moveable arm troughs. Once the participant was aligned to the middle of the seat, the robotic exoskeleton was adjusted to align the elbow and shoulder to the joints of the robotic linkage, permitting arm movements in the horizontal plane. Seat height was adjusted such that each participant was in ~ 80–85 degrees of shoulder abduction. A virtual reality system aligned with the horizontal workspace displayed spatial targets and a cursor directly representing the position of the individual’s index finger (white dot, 0.5 cm radius).

Participants were required to move the white dot to a red center target (1-cm radius) and make a 10-cm reaching movement to 1 of 8 peripheral red targets (1-cm radius) once they appeared [13]. Upon peripheral target illumination, participants had 3000 ms to complete the reaching movement, with movement offset identified once the participant reached the peripheral target with their white dot. Movement offset was calculated by either (1) the first local minimum in hand speed that is below a calculated upper speed threshold or (2) when the hand speed dropped below a calculated lower speed threshold. These thresholds have been described in-depth previously [13]. If participants were unable to reach the target within 3000 ms, this was identified as a failed trial by Kinarm’s algorithm. Further, movement overshoot/undershoot to the peripheral target was not sufficient to produce a successful reaching trial, with participants being required to stabilize their cursor within the peripheral target. Participants were then required to move the white dot back to the center target and hold once again after the peripheral target disappeared. Participants were instructed by the Kinarm operator to make the reaching movement as quicky and accurately as possible. Peripheral target presentation was pseudorandomized within a block, and participants were required to make 8 movements to each of the 8 peripheral targets, for a total of 64 reaching movements. Participants performed the task with both their ipsilesional and contralesional arm, with the order of the arm assessed randomly selected. Movies demonstrating a participant completing the VGR task can be found in Additional files 8 and 9.

The VGR task and its measurement parameters have been previously described [13, 23, 24]. The ability of the task to discriminate performance between the limbs has been well established [13]. Measurements of upper extremity impairment in the present paper focused on 3 individual parameters and an overall task score developed from 9 available parameters recorded by the Kinarm [13, 23, 24]. The 3 parameters were selected for their ability to assess impairments when reacting to visual stimuli and making accurate and efficient reaching movements in response:

- Reaction time—The time between a peripheral target appearing and the participant initiating a reaching movement out to it. This variable is measured in milliseconds and assesses impairments in visuomotor response time.
- Initial direction error—The amount of angular error between an ideal straight-line movement from the center target to a peripheral target and the participant’s recorded movement trajectory. This variable is measured in degrees and assesses impairments in trajectory control during reaching movements.
- Movement time—The total time required to complete a movement from the center target to a peripheral target. This variable is measured in seconds and measures impairments in arm motor coordination.

Task parameters were transformed into standardized z-units using a model of neurological healthy control participants [25] (For details, see https://kinarm.com/kinarm-products/kinarm-standard-tests). The distribution of task parameter values from these control individuals were transformed into a standard normal distribution (mean = 0, standard deviation = 1) using Box-Cox equations with linear regressions to consider the influence of sex, handedness, and age on performance. A participant with stroke was considered impaired on a parameter if their z-score fell outside of the 95% performance range of healthy controls (z > 1.64) (Kinarm Standard Tests, Kinarm, Kingston, ON, Canada).

To quantify overall performance on the VGR task, a z-Task Score was also calculated for each participant at every time point using 9 parameters captured by the Kinarm (see Additional file 1: Methods for the 6 parameter descriptions not listed above). A description of this mathematical process has been described previously [25] (For details, see https://kinarm.com/kinarm-products/kinarm-standard-tests). These z-Task Scores used a model derived from neurologically healthy individuals (n = 321, aged 18–84 years old) to transform the root-sum squares of these nine task parameter values into a standard normal distribution. Z-Task Scores > 1.64 were identified as impaired (95th percentile of healthy individual performance). This classification was additionally used to determine impairment recovery of the ipsilesional and contralesional arms post-stroke, with arms recording z-Task Scores ≤ 1.64 at 6-months post-stroke being labelled as recovered.

### Clinical assessments

At each of the 4 time points, participants were assessed for arm and hand motor impairment (Chedoke-McMaster Stroke Assessment (CMSA) [26]), dexterity (Purdue Pegboard (PPB) [27]), functional ability (Functional Independence Measure (FIM) [28]), and visuospatial neglect (Behavioural Inattention Test (BIT) [29]). The CMSA is scored from 1–7 for both the hands and the arms, with 1 representing the lowest level of functioning and 7 representing the highest level of functioning [26]. We chose the CMSA as it is commonly used in Canadian rehabilitation centres to quantify limb impairment. The PPB measures how many metal pegs can be placed into cylindrical holes in 30 s [27]. Age-matched normative data in healthy individuals is readily available (Bolla-Wilson & Kawas [30]). The FIM is scored out of 126, with a score of 18 representing the lowest level of functioning and a score of 126 representing the highest level of functioning [28]. For this study, the conventional sub-tests of the BIT were used, including line crossing, letter cancelation, star cancellation, figure and shape copying, line bisection, and representational drawing. A score of less than 130 was considered impaired for the BIT [29] and is thought to be consistent with visuospatial neglect. All assessments were performed by a trained therapist or physician (Table 1).

**Table 1:** Participant demographics and clinical assessments

Parameters	Final selected participants (n = 106)
Age (Years—Mean ± SD)	59.5 ± 13.9
Sex	73 M/33 F
Handedness	6 L/100 R
Type of Stroke	85 I/21 H
Side of Lesion	44 L/62 R
Days Since Stroke^a^ (Mean ± SD)	12.1 ± 8.8
CMSA^b^ [1–7]^d^	
Contralesional Arm – 1-Week Post-Stroke	[8, 12, 13, 17, 19, 22]
Contralesional Arm – 6-Weeks Post Stroke	[3,11,11,7,12,16,46]
Contralesional Arm – 12-Weeks Post-Stroke	[2,5,11,5,12,21,50]
Contralesional Arm – 26-Weeks Post-Stroke	[0,2,11,8,8,13,64]
Ipsilesional Arm – 1-Week Post Stroke	[0,0,0,1,1,15,86]
Ipsilesional Arm – 6-Weeks Post-Stroke	[0,0,0,0,0,5,101]
Ipsilesional Arm – 12-Weeks Post-Stroke	[0,0,0,0,0,5,101]
Ipsilesional Arm – 26-Weeks Post-Stroke	[0,0,0,0,0,3,103]
CMSA^c^ [1–7]^d^	
Contralesional Hand – 1-Week Post-Stroke	[7, 9, 11, 15–17, 27]
Contralesional Hand – 6-Weeks Post-Stroke	[5, 7, 8, 21, 26, 34]
Contralesional Hand – 12-Weeks Post-Stroke	[3,6,7,4,17,27,42]
Contralesional Hand – 26-Weeks Post-Stroke	[1,5,7,4,15,24,50]
Ipsilesional Hand – 1-Week Post-Stroke	[0,0,0,0,0,30,72]
Ipsilesional Hand – 6-Weeks Post-Stroke	[0,0,0,0,0,15,91]
Ipsilesional Hand – 12-Weeks Post-Stroke	[0,0,0,0,0,14,92]
Ipsilesional Hand – 26-Weeks Post-Stroke	[0,0,0,0,0,8,98]
FIM ^e^ (Mean ± SD)	
FIM – 1-Week Post-Stroke	92.8 ± 23.9
FIM – 6-Weeks Post-Stroke	112.4 ± 14.7
FIM – 12-Weeks Post-Stroke	118.0 ± 10.3
FIM – 26-Weeks Post-Stroke	120.8 ± 7.9
BIT ^f^ (Mean ± SD)	
BIT – 1-Week Post-Stroke	130.9 ± 24.7
BIT – 6-Weeks Post-Stroke	140.0 ± 8.0
BIT – 12-Weeks Post-Stroke	141.0 ± 6.6
BIT – 26-Weeks Post-Stroke	141.9 ± 4.9
PPB^g^ (Mean ± SD (# of Participants that Placed 0 Pegs))	
Contralesional Hand – 1-Week Post-Stroke	5.8 ± 4.7 (32)
Contralesional Hand – 6-Weeks Post-Stroke	7.9 ± 4.7 (19)
Contralesional Hand – 12-Weeks Post-Stroke	8.5 ± 4.8 (17)
Contralesional Hand – 26-Weeks Post-Stroke	8.8 ± 4.6 (15)
Ipsilesional Hand – 1-Week Post-Stroke	7.6 ± 4.7 (19)
Ipsilesional Hand – 6-Weeks Post-Stroke	9.4 ± 4.4 (11)
Ipsilesional Hand – 12-Weeks Post-Stroke	10.1 ± 4.2 (7)
Ipsilesional Hand – 26-Weeks Post-Stroke	10.4 ± 4.1 (6)

^a^Three participants missed 1-week assessment; 6-week assessment used instead. ^b^Chedoke-McMaster Stroke Assessment arm scores were not captured for 3 participants at 1-week assessment. ^c^Chedoke-McMaster Stroke Assessment hand scores were not captured for 4 participants at 1-week assessment. ^d^Bracketed scores in second column represent number or participants at each score on the CMSA, corresponding with position in first column. ^e^Functional Independence Measure scores were not captured for 3 participants at 1-week assessment. ^f^Behavioural Inattention Test scores were not captured for 3 participants at 1-week assessment. ^g^Purdue Pegboard Test scores were not captured for 3 participants at 1-week assessment

### Imaging

Clinical MRI or CT scans (73 MRI, 27 CT) were collected for 100 participants at the time of stroke (2.6 ± 3.7 days post-stroke). We were unable to obtain scans for some participants (n = 6) as their imaging was done outside of our centre. Imaging was collected using either a General Electric 1.5-T or 3.0-T MRI scanner, or a Siemens System CT scanner. Imaging acquisition sequences were conducted according to acute stroke protocols at the Foothills Medical Centre. Imaging included T2 fluid-attenuated inversion recovery, diffusion-weighted imaging, and CT scans. These clinical scans were used to determine stroke lesion volume. Further information regarding imaging can be found in the Additional file 1: Methods.

### Data and statistical analyses

All statistical analyses were performed with the Statistics and Machine Learning Toolbox offered in the 2021a version of MATLAB (MathWorks, Natick, Massachusetts, USA). One-sided Wilcoxon rank sum tests were used to test for differences in both CMSA scores and PPB scores between the ipsilesional and contralesional arms. Linear Mixed-Effects Models (LMMs) were used to test for differences in robotic measures of ipsilesional and contralesional arm impairment, determining the effects side of lesion and time post-stroke had on impairment severity. To test assumptions of normality in the distributions of our data, residuals from LMMs were visualized in a bar plot with a normal distribution curve fitted over top using the “histfit” function contained within the 2021a version of MATLAB. Bonferroni adjustments were used to correct for multiple comparisons in our LMMs. One-way analyses of variance were used to identify significant fixed effects from LMMs on impairment severity. Two-by-two Chi-Squared tests were used to determine if the number of statistically significant z-score changes we observed was due to chance or not. Linear regressions were used to determine the relationship between robotic measures of ipsilesional and contralesional arm impairment. Two-sample Kolmogorov–Smirnov tests were used to test for distribution differences in correlation analyses of robotic measures of impairment, accounting for side of lesion. An unpaired t-test was used to compare lesion volume between participants with left- and right-hemisphere damage. Additional details regarding the statistical methods utilized can be found in the Additional file 1: Methods section.

## Results

We assessed 106 participants (73 male/33 female) at four separate time points in the first 6-months post-stroke (see Table 1). The average age of the participants assessed was 59.5 ± 13.9 years, and 100 participants were right-handed. Of the 106 participants, 85 had experienced a unilateral ischemic stroke, and the remaining 21 had experienced a unilateral hemorrhagic stroke. Sixty-two strokes occurred in the right-hemisphere of the brain, and the remaining 44 strokes occurred in the left-hemisphere. When grouped by lesioned hemisphere, there was no statistically significant difference between right- and left-hemisphere lesion volume (h = 0, p = 0.264). At the first time point, twenty-four participants (20.9%) had visuospatial neglect, as determined by the BIT.

Examining the CMSA at the first time point revealed that 17 participants (16.0%) had ipsilesional arm impairments whereas 81 participants (76.4%) had contralesional arm impairments. Additionally, 30 participants (28.3%) had ipsilesional hand impairments whereas 86 participants (81.1%) had contralesional hand impairments at the same time point. Unsurprisingly, one-sided Wilcoxon rank sum tests revealed that the ipsilesional arm scored significantly higher on the CMSA at each of the 4 time points for both the arm and hand (p < 0.001 for all time points for both the arm and hand), indicating better performance. When examining the PPB, we found that the ipsilesional arm placed significantly more pegs than the contralesional arm at all 4 time points (p < 0.05 for all time points). We also compared the number of pegs placed to normative data taken from Bolla-Wilson & Kawas [30] on the PPB. When matched by age, sex, and handedness with normative controls, stroke participants placed significantly less pegs with both their ipsilesional and contralesional arm at 1-week post-stroke (p < 0.001). We repeated this comparison using PPB scores from 6-months post-stroke and found the same results (p < 0.001). Details of these comparisons can be found in the Additional file 1: Methods section. Although not assessed as a primary outcome measure for this study, a summary of participant scores on the Modified Ashworth Scale for each of the four time points can be found in Additional file 7: Table S6.

### Robotic assessments of ipsilesional arm impairment

Figure 1 demonstrates exemplar reaching movements on the VGR task for a healthy, neurologically-intact control participant (Fig. 1B), and a participant with a right-hemisphere stroke (Fig. 1C–F). At 1-week post-stroke (Fig. 1C), the participant’s movements often failed to reach the end target with their contralesional arm. The stroke participant’s ipsilesional arm movements had reduced speed (13.1 cm/s versus normative range of 16.1–39.7 cm/s) and an increased number of corrective movements at 1-week post-stroke. By 6-months post-stroke (Fig. 1F), the participant’s reaching movements in both arms appeared closer to those of the control. However, impairments, primarily in the contralesional limb, were still present with an increased number of corrective movements and angular deviations from straight-line reaching movements (speed maxima count of 3.05 versus normative range of 1.86–2.98, path length ratio of 1.37 versus normative range of 1.06–1.26).

**Fig. 2:**
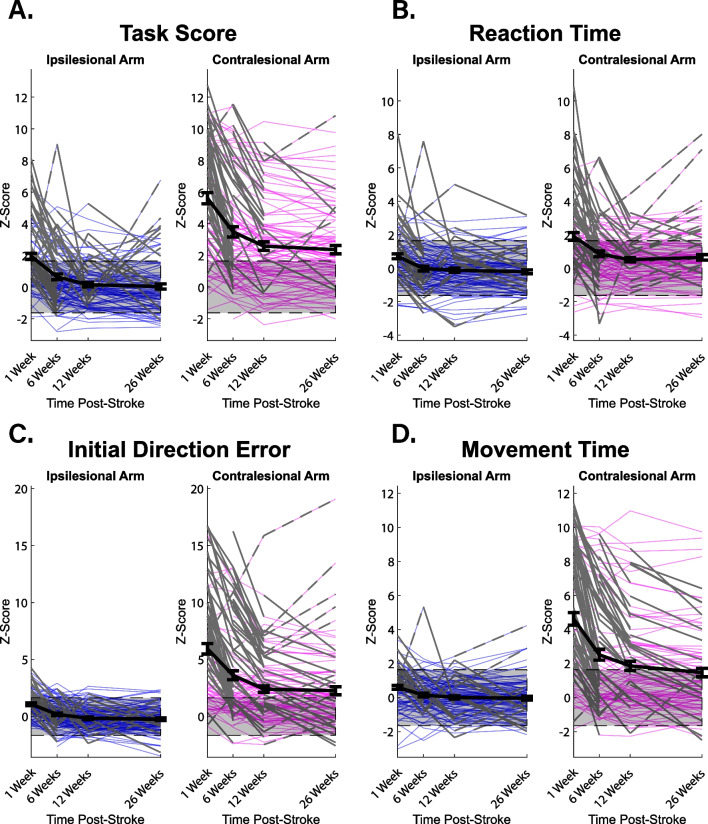
Line plots demonstrating individual performance of all 106 participants on 4 separate parameters of the visually guided reaching task: **A** Z-Task Score, **B** Reaction Time, **C** Initial Direction Error, and **D** Movement Time. Thick black lines represents mean z-score with standard error of the mean at each time point. Thin solid grey lines represent a significant improvement in z-score from one time point to the next. Thin dashed grey lines represent a significant worsening in z-score from one time point to the next. Grey shaded regions represent normative score ranges for each parameter

On the VGR task, we found that 47.1% of participants had significant impairments in their ipsilesional arm 1-week post-stroke, as determined by a z-Task Score > 1.64. Additionally, 83.5% of participants had significant impairments in their contralesional arm at the same time point, determined by the same measure. Figure 2 demonstrates individual performance of all 106 participants on 4 selected parameters of the VGR task. As highlighted in Fig. 2A, the mean ipsilesional arm z-Task Score (black line) was lower (signifying better performance) than the mean contralesional arm z-Task Score at each of the 4 time points. A similar relationship can be seen for the three remaining parameters presented in Fig. 2, but the differences between the mean contralesional arm scores and ipsilesional arm scores varied between parameters (see Fig. 2B–D).

We used LMMs to examine the impact of fixed (time point, arm status, side of lesion), and random (individual participant performance) effects on mean z-scores. We found significant differences for the overall z-Task Scores when accounting for arm status (ipsilesional versus contralesional) and time point (p = 1.87 × 10^–24^, p = 3.68 × 10^–22^, respectively). This meant the ipsilesional arm z-Task Scores were significantly lower (better) than the contralesional arm z-Task Scores, and that the scores improved with time. Pairwise comparisons of these two fixed effects further confirmed this difference in scores between the ipsilesional and contralesional arm, providing a statistical measure of the significant difference in mean z-Task Score from the ipsilesional and contralesional arm at each of the 4 time points. However, pairwise comparisons of the fixed effects of our LMM revealed that the change in z-Task Score for both the ipsilesional and contralesional arm was not significant from 12- to 26-weeks post-stroke, and that the change in z-Task Score for the ipsilesional arm from 6- to 12-weeks post-stroke was not significant. Significant differences in arm status, time point, and their interactions were reflected in the remaining 3 parameters shown in Fig. 2, save for the interaction between arm status and time point for reaction time: reaction time (p = 1.63 × 10^–8^, p = 1.30 × 10^–11^, p = 0.368, respectively), initial direction error (p = 3.19 × 10^–31^, p = 5.42 × 10^–21^, p = 4.05 × 10^–5^, respectively), and movement time (p = 299 × 10^–38^, p = 1.20 × 10^–25^, p = 1.91 × 10^–9^, respectively). All LMM results can be found in Additional file 2: Table S1. Also, all coefficients and their resulting p-values from the LMMs can be found in Additional file 3: Table S2.

When examining the fixed effect of side of lesion, we found there were no significant differences in z-Task Score when side of lesion was assessed alone (p = 0.561), assessed in combination with time point (p = 0.738), or assessed in combination with arm status (p = 0.442). First, this meant that there were no significant differences in z-Task Score as a whole from both arms when grouped by side of lesion. Second, this meant that when z-Task Score was grouped by side of lesion, there were no significant differences in z-Task Score at any of the 4 time points. And third, this meant that when z-Task Score was grouped by side of lesion, there were no significant differences in impairments for participants with either left or right hemisphere damage in either the contralesional or ipsilesional arm. We repeated this analysis without our 6 left-handed participants and found similar results. We then went on to examine individual parameters using the whole sample (n = 106) and observed no significant difference between participants with left- or right-hemisphere damage in initial direction error (p = 0.253, p = 0.483, p = 0.368, respectively), movement time (p = 0.089, p = 0.589, p = 0.088, respectively), and reaction time (p = 0.865, p = 0.146, p = 0.244, respectively). All results of our LMM examining the impact of stroke-lesioned hemisphere on performance can be found in Additional file 2: Table S1. We also assessed the impact of neglect on our LMM results, but found no significant differences for any of our parameters when neglect was factored in as a fixed effect (Additional file 6: Table S5). Across the 4 parameters shown in Fig. 2, the majority of all significant improvements in individual z-scores occurred in the 1- to 6-week post-stroke period for both the ipsilesional and contralesional arms: z-Task Score (66.0% and 66.1%, respectively), reaction time (65.0% and 69.4%, respectively), initial direction error (65.6% and 49.4%, respectively), and movement time (50.0% and 53.8%, respectively). Significant changes for all parameters across all time points are presented in Additional file 4: Table S3. It is important to note that while some participants continued to make significant improvements over the entire 6-months of our observation period, others had their performance worsen or stabilize with time (see contralesional arm in Fig. 2B, C). We examined whether the individual changes in each parameter that occurred between time points observed could be due to chance using Chi-squared tests. The analysis revealed that even in the 12- to 26-week time period, 50% of parameters measured were changing beyond what would be expected by chance alone (see Additional file 4: Table S3).

**Fig. 3:**
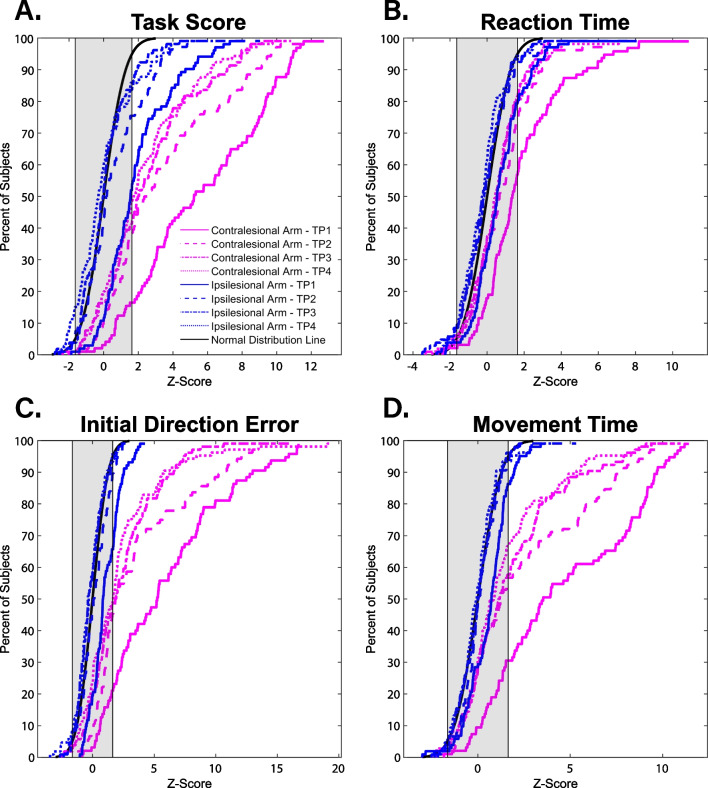
Cumulative sum histograms the proportion of participants that had normative scores for selected parameters of the visually guided reaching task at each of the 4 time points: **A** Z-Task Score, **B** Reaction time, **C** Initial Direction Error, and **D** Movement Time. Shaded regions indicate normative scores for each parameter. Normal distribution lines were generated for each parameter form − 3 to 3 at steps of 0.01

To assess the proportion of participants that performed within the normative range of scores on the VGR task in the first 6-months post-stroke, cumulative sum histograms were generated for the parameters of the VGR task for both the ipsilesional and contralesional arm. Figure 3 highlights these parameters (z-Task Score, reaction time, initial direction error, and movement time), providing the percent of participants that were within the normative range of z-scores for each parameter. For z-Task Score (Fig. 3A), the percentage of participants with ipsilesional arm performance that fell within the normal range, as determined by a z-Task Score < 1.64, went from 52.9% to 85.8% over the 6-month assessment period. The percentage of participants with contralesional arm performance that fell within the normal range, also determined by a z-Task Score < 1.64, went from 16.5% to 48.6% over the same time period. Notably, at 6-months post-stroke, 15.1% of participants had ipsilesional arm z-Task Scores below -1.64, denoting excellent performance (Fig. 3A).

**Fig. 4:**
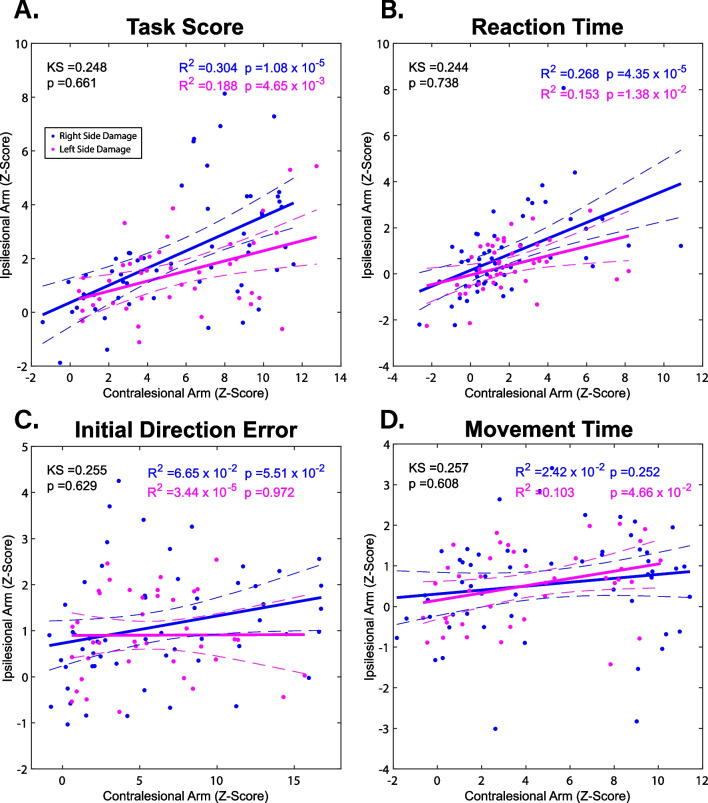
Linear regressions comparing scores of the contralesional and ipsilesional arms for selected parameters of the visually guided reaching task at the first time point, separated by side of lesion: **A** Z-Task Score, **B** Reaction time, **C** Initial Direction Error, and **D** Movement time. Solid blue regression lines are for right side damage participants, solid magenta regression lines are for left side damage participants. Dashed lines represent the 95% confidence intervals of the linear regressions, matching for colour. KS represents the results of 2-sample Kolmogorov-Smirnov tests run for each parameter with resulting p-values

To determine if (1) the severity of contralesional arm impairments predicted the severity of ipsilesional arm impairments and (2) whether the side of lesion had an impact on this prediction, linear regressions were completed for the parameters of the VGR task, with data grouped by side of lesion. Figure 4 shows these parameters (z-Task Score, reaction time, initial direction error, and movement time), plotting z-scores of the contralesional and ipsilesional arms against one another at the first time point for each participant. Many parameters demonstrated strong significant relationships between the contralesional and ipsilesional arm at 1-week post-stroke, with a low to moderate amount of variance explained (R^2^). For example, the relationships for z-Task Score (Fig. 4A) and reaction time (Fig. 4B) were significant for both the right side lesion (RSL) (blue) and left side lesion (LSL) (magenta) regression, but had the following coefficients of determination (R^2^ = 0.304 & 0.188 and R^2^ = 0.268 & 0.153, respectively). Other parameters demonstrated an insignificant relationship for both regressions (e.g., Initial Direction Error, Fig. 4C). For each parameter, two-sample Kolmogorov–Smirnov tests were used to determine whether the RSL and LSL regressions differed from one another significantly. All two-sample Kolmogorov–Smirnov tests run failed to reject the null hypothesis, indicating no impact of side of lesion on whether ipsilesional arm impairments were related to the severity of contralesional arm impairments.

We went on to examine whether the severity of contralesional arm impairments predicted the severity of ipsilesional arm impairments at 6-months post-stroke. Thus, we repeated the linear regressions with data from 6-months post-stroke. For the 4 parameters shown in Fig. 4, strong significant relationships were again found between the contralesional and ipsilesional arm in some parameters. The reaction time parameter had the strongest relationship at 6-months post-stroke (p = 6.06 × 10^–10^ & R^2^ = 0.480 (RSL), p = 2.77 × 10^–7^ & R^2^ = 0.470 (LSL)). This was followed by the movement time parameter (p = 1.46 × 10^–4^ & R^2^ = 0.218 (RSL), p = 5.98 × 10^–5^ & R^2^ = 0.322 (LSL)) and the z-Task Score parameter (p = 4.67 × 10^–5^ & R^2^ = 0.247 (RSL), p = 4.86 × 10^–2^ & R^2^ = 0.089 (LSL)). All Kolmogorov–Smirnov test results are presented in Additional file 5: Table S4. We again found no statistically significant differences between the RSL and LSL regressions at 6-months post-stroke, as determined by Kolmogorov–Smirnov tests.

## Discussion

In our study, we found that the ipsilesional arm was significantly impaired in nearly half of the participants studied when assessed through robot-based measures at 1-week post-stroke. By 6-months post-stroke, the proportion of ipsilesional arm impairments had decreased to 14.2%, while many individuals still had impairments in the contralesional arm (51.4%). We also found that while the nature and severity of ipsilesional arm impairments varied between individuals, significant relationships were observed with the severity of contralesional arm impairments at both 1-week and 6-months post-stroke in several robotic parameters (Fig. 4 and Additional file 5: Table S4). Finally, we found that side of lesion had no impact on the severity of impairments in either arm (Fig. 4, Additional file 2: Table S1 and Additional file 5: Table S4).

### Robotic and clinical assessments of ipsilesional arm motor impairment in the first 6-months post-stroke

Unsurprisingly, both the PPB and the CMSA revealed ipsilesional arm motor impairments were less severe than those of the contralesional arm [6, 31]. Despite the lesser severity of ipsilesional motor impairments, they are still prevalent, and some authors have suggested they should not be overlooked with rehabilitation [32]. If the contralesional limb fails to recover following stroke, the ipsilesional limb becomes the dominant limb required to complete activities of daily living [9], and if this limb is impaired, these activities become increasingly difficult to complete [10].

While there was overlap in the participants identified as having had ipsilesional arm impairments at 1-week post stroke by the arm portion of the CMSA and the VGR task, the overall VGR task score identified 31 more participants as having had ipsilesional arm impairments at 1-week post-stroke (Table 1, Fig. 2). It is unfortunate that a commonly used clinical scale, such as the CMSA, is unable to detect these motor impairments. However, the PPB was able to provide further insight on motor impairments, with participants placing significantly less pegs than normative age-, sex-, and handedness-matched controls at both 1-week and 6-months post-stroke with both their ipsilesional and contralesional arm. The increased sensitivity of the robotic assessment captured subtler impairments related to the quality of movement, a feature that could allow clinicians to prescribe precise, impairment-specific rehabilitation on a patient-by-patient basis in the future [13, 33, 34].

### Improvements in arm motor impairments over the first 6-months post-stroke

Our findings regarding motor recovery in the first 6-months post-stroke differ from previous work in the literature. A recent study by Cortes et al. [35] in a smaller sample of participants (n = 18) using a similar robotic task suggested a plateau in motor recovery for both the ipsilesional and contralesional arm at 5-weeks post stroke. When our data was assessed at the group level, pairwise comparisons of the fixed effects from our LMM revealed a plateau in motor recovery for the ipsilesional arm by 6-weeks post-stroke but showed contralesional arm motor performance significantly improved up to 12-weeks post-stroke.

When we examined individual participant performance, however, we found evidence that VGR task scores commonly changed from 12- to 26-weeks post-stroke, with some participants improving, and others deteriorating. Established thresholds for the VGR task [25] revealed that over 10% of all individual participant z-score changes between 12- and 26-weeks post-stroke were significant for several parameters (reaction time, initial direction error, movement time, see Additional file 4: Table S3). We confirmed that these results were not due to chance. Deteriorations in performance are likely the result of decreased arm use in daily life, cessation of active rehabilitation, or a combination of both [36]. These individual differences suggest that motor impairments may continue to change beyond what some previous literature has historically proposed based on clinical measures [3, 5], and further supports the use of more sophisticated equipment like the Kinarm to detect subtle motor impairments missed by clinical measures [38].

### Relationship between ipsilesional and contralesional arm motor impairments

In line with findings from previous studies [18, 19], we observed that for most parameters of the VGR task, there was a statistically significant relationship between ipsilesional and contralesional upper-extremity performance (Fig. 4). However, in the work done by Varghese and Winstein [19], this relationship was found only in partipants with unilateral left-hemisphere stroke, and not in right-hemisphere stroke participants. Our current study found no hemispheric differences for this relationship at either 1-week or 6-months post-stroke. Although the Varghesse & Winstein study [19] assessed hand function specifically, a clinical assessment for the whole arm (Upper Extremity Fugl-Meyer) was used to examine contralesional motor impairments, while a different assessment which places much emphasis on the hand (Wolf-Motor Function Test) was used to examine ipsilesional motor impairments. In comparison, our study utilized an identical assessment to measure arm impairment for both the ipsilesional and contraleisonal limbs, which may explain why our study did not observe the hemisphere-specific impairments reported in previous work [19].

Our study found that when motor impairments were compared by stroke-lesioned hemisphere, there were no significant differences in impairment type or severity for any parameter assessed (Fig. 4, Additional file 2: Table S1 and Additional file 3: Table S2). Previous work has found hemispheric differences when looking at ipsilesional arm motor impairments, with left-hemisphere damage causing issues with trajectory direction and curvature in reaching movements and right-hemisphere damage causing issues with movement impedence control [14, 17]. Unlike our work, these studies were both conducted with less than 15 participants who were all right handed with chronic stroke. Even though our study calculated movement trajectory in a similar manner to these studies (angular deviation from a straight-line path, most similar to Schaefer, Haaland, & Sainburg’s 2009 study), we were unable to replicate the hemispheric differences seen in previous work in our current study. However, the 2007 study [14] utilized a 2-target reaching task that participants completed without visual feedback, which may have produced endpoint errors, compared to our 8-target task which provided a visual target to guide movement [14]. Providing visual feedback during the task in our study may explain the differences we observed, and make it more difficult to compare endpoint control between our work and Schaefer et al. 2009 study. Recent studies examining hemispheric differences in ipsilesional motor impairments have also shown that side of lesion has no significant impact on the type or severity of motor impairments [6, 20], the largest of which had data from 227 stroke survivors [8].

## Limitations

As with other clinical work, our data had missing points due to participant dropout and inability to attend sessions (1.74% of the total sample of assessments). Additionally, we had only a few participants with severe stroke, and only 6 left-handed participants. Also, the individual significant change thresholds used were established through repeat testing of healthy controls [25], and higher variance between repeated testing may be present in stroke survivors. We did not collect the Fugl-Meyer Assessment, which has been used in many of the previous studies on the ipsilesional arm. However, CMSA is highly correlated with the Fugl-Meyer Assessment (r = 0.95) [26]. Further, although the Modified Ashworth Scale was used to assess spasticity in our cohort and spasticity was typically absent or very mild in our subacute stroke group we did not use the MAS scores to stratify performance on our robotic assessments of motor impairment. Finally, the robotic exoskeleton used for this work supported the arms of stroke participants from gravity, which is different from some [10, 32], but not all previous methods [8, 14, 37] used to assess ipsilesional arm motor impairments.

## Conclusion

We demonstrated the variable nature of ipsilesional arm motor impairments, and quantified their recovery over the first 6-months post-stroke. We also showed that ipsilesional arm impairment is related to the severity of contralesional arm impairment, but this relationship depended on the robotic parameter measured. Additionally, we addressed a common debate in the literature surrounding hemisphere-specific impairments, showing that side of lesion was not related to the type or severity of motor impairments seen in either the ipsilesional or contralesional arm in our reaching task. Finally, the high proportion of participants that performed normally with their ipsilesional arm at 6-months post-stroke begs the question of whether ipsilesional arm rehabilitation may only need to be considered in rarer cases where ipsilesional impairments are severe and slow to recover (Additional file 8 and Additional file 9).

### Supplementary Information

**Additional file 1. **Methods

**Additional file 2. Table S1.** Results of the ANOVA completed for the fixed effects of the linear mixed models for each of the 4 parameters of the VGR task. Bolded values indicate statistical significance at the 95% confidence level. FStat: F-statistic of the ANOVA test used for the linear mixed model.

**Additional file 3. Table S2.** Estimates and corresponding p-values for all fixed effects of the linear mixed models for each parameter. Bolded values indicate statistical significance at the 95% confidence level. Bracketed values indicate the lower and upper bounds of each estimate, respectively. Ipsi: Ipsilesional Arm, LSL: Left-side lesion, TP: Time Point. Asterix indicates an interaction between fixed effects.

**Additional file 4. Table S3.** Significant changes in parameter scores from one time point to the next for all parameters presented. Bolded values indicate statistical significance of Chi-squared test at the 95% confidence level. Brackets: (Significant improvement in score with contralesional arm, significant degradation in score with contralesional arm, significant improvement in score with ipsilesional arm, significant degradation in score with ipsilesional arm). Chi: Chi-square test value.

**Additional file 5. Table S4.** Results of the linear regressions comparing the effect of left-versus-right side lesions on impairment severity for the ipsilesional and contralesional arm for all parameters presented. Bolded values indicate statistical significance at the 95% confidence level. LSL: Left-side lesion, RSL: Right-side lesion, KS: Kolmogorov-Smirnov test value.

**Additional file 6. Table S5.** Results of the ANOVA completed for the fixed effects of the linear mixed models for each of the 4 parameters of the VGR task with neglect accounted for. Bolded values indicate statistical significance at the 95% confidence level. FStat: F-statistic of the ANOVA test used for the linear mixed model.

**Additional file 7. Table S6.** Summary of participant scores on the Modified Ashworth Scale for the contralesional arm at each of the four time points. Bracketed numbers represent the number of participants at each score at each time point. Modified Ashworth Scale scores follow the following order in brackets: (0, 1, 1+, 2, 3, 4). Modified Ashworth Scale was not collected for 13 participants at 1-week post-stroke, 7 participants at 6-weeks post-stroke, 9 participants at 12-weeks post-stroke, and 3 participants at 26-weeks post-stroke.

**Additional file 8. **Visually guided reaching—Left Arm (Contralesional).

**Additional file 9. **Visually guided reaching—Right Arm (Ipsilesional).

## Data Availability

Data available upon request.

## Abbreviations

VGR: Visually guided reaching
CMSA: Chedoke McMaster Stroke Assessment
FIM: Functional independence measure
BIT: Behavioural Inattention Test
PPB: Purdue Pegboard
LMM: Linear Mixed Model
RSL: Right side lesion
LSL: Left side lesion
