# Supplementary material for: Impairments of the ipsilesional upper-extremity in the first 6-months post-stroke
Source: J Neuroeng Rehabil. 2023 Aug 14;20:106. doi: 10.1186/s12984-023-01230-8 (PMC10424459; doi:10.1186/s12984-023-01230-8)
Supplement: Supplementary file 1 — Additional file 1. Methods [file 12984_2023_1230_MOESM1_ESM.docx]

Additional file 1: Methods

*Purdue Pegboard Test – Comparison to Normative Values*

To compare our experimental cohort’s Purdue Pegboard (PPB) Test scores to normative age and sex matched controls, we pulled values from a study by Bolla-Wilson & Kawas (30). For each age bin presented in the cited study, we pulled PPB scores from our cohort and compared, matching for age, sex, and handedness. For our cohort, if a participant’s contralesional arm was their dominant arm pre-stroke, their PPB scores were compared against the dominant hand normative control scores presented by Bolla-Wilson and Kawas; if our participant’s contralesional arm was their non-dominant arm pre-stroke, they were compared with non-dominant normative control scores. This was completed for PPB scores recorded at both 1-week and 6-months post-stroke. As the cited work only had normative control data for participants 40-89 years old, we were unable to compare data for 10 of our participants, all of which were less than 40 years of age at presentation. To compare experimental and control PPB scores, we ran paired 2-sample t-tests for the contralesional and ipsilesional arm at both 1-week and 6-months post-stroke.

*Kinarm Parameters Not Listed in Manuscript Used to Calculate Z-Task Score*

- Posture Speed – Hand speed recorded while the participant held their hand at the center target.
- Initial Distance Ratio – The ration of initial to full movement length made by a participant during a reaching movement.
- Speed Max Count – The number of speed peaks recorded in a movement from the center target to a peripheral target.
- Minimum-Maximum Speed Difference – The mean difference between pairs of local speed maxima and minima recorded during a reaching movement.
- Path Length Ratio – The ratio of the total recorded distance of a participant’s reaching movement from the center target to a peripheral target to an ideal straight-line movement between the two.
- Maximum Speed – The peak hand speed recorded during a reaching movement from the center target to a peripheral target.

*Imaging Methods*

Imaging slice thickness varied from 3.0 mm to 5.0 mm, depending on the scanner type, all with at least a 1.0 mm x 1.0 mm in-plane resolution. All stroke lesions were manually identified and marked using MRIcron^1^ by trained assessors on each participant’s fluid-attenuated inversion recovery (FLAIR) or CT scans. If available, diffusion-weighted imaging was used to help assist lesion identification and marking. Lesion markings were then verified by a stroke neurologist. From these markings, a volume of interest (VOI) was created for each participant. Lesions volume metrics were all calculated in subject-space. To compare lesion volume size between participants with left- and right-hemisphere strokes, an unpaired t-test was used.

*Movement Onset Determination in the Kinarm Robotic Exoskeleton*

To identify when movement onset occurred for each reach of the VGR task, 2 statistical thresholds were first calculated for each participant while they held their hand at the center target: 1) lower speed threshold – median hand speed recorded across all trials during the 500 ms prior to illumination of a peripheral target, and 2) upper speed threshold – the 95^th^ percentile of recorded hand speed during this same time period.^2^ When making a reaching movement, the algorithm used by the Kinarm identified when the participant’s hand left the center target in response to a peripheral target appearing. The algorithm then moved backwards in time to find the first instance of either 1) a local minimum in hand speed that was below the calculated upper speed threshold, or 2) the point in which hand speed dropped below the lower speed threshold.^2^ To identify movement offset, the algorithm identified the point when the participant’s hand entered the peripheral target and found either 1) the first local minimum in hand speed that was below the upper speed threshold, or 2) the point in which hand speed dropped below the lower speed threshold.

*Significant Change Threshold Determination for the VGR Task*

Recent work has established thresholds for the VGR task to determine when a significant change in z-score occurs from one assessment to the next (3). These thresholds were calculated by first testing 50 healthy controls on the VGR task twice, determining the standard deviation in their z-scores, and establishing a confidence interval from the standard deviation values. From this, significant changes in z-score for a parameter of the VGR task were calculated by multiplying the square root of 2 by the confidence interval for said parameter.^3^ Using these significant change thresholds for the VGR task, significant changes in z-score, both improvements and declines, were plotted for the parameters of the VGR task for all participants (see Figure 2). This allowed us to determine when participants made the majority of their improvements in VGR task performance in the first 6-months post-stroke.

*Missing Data*

Out of 424 assessment timepoints, we had 16 missing datapoints from the VGR task that came from 13 participants. At the 1-week post-stroke assessment, 5 participants were unable to complete the task with their contralesional arm due to the severity of their motor impairments, and 4 participants missed the assessment entirely. At the 6-week post-stroke assessment, 1 participant was unable to complete the task with their contralesional arm due to the severity of their motor impairments, and another participant had missing data from their contralesional arm due to an error with downloading task the task report from the Kinarm. At the 12-week post-stroke assessment, 1 participant was unable to complete the task with their contralesional arm due to the severity of their motor impairments, 1 participant had missing data from both arms, and 1 participant had missing data from their ipsilesional arm (both missing data cases due to errors with downloading task reports from the Kinarm). At the 26-week post-stroke assessment, 1 participant experienced a robot calibration error with their contralesional arm while performing the task, and no data was recorded as a result.

*Linear Mixed Model Setup and Verification*

All linear mixed models were calculated using the following equation: ‘VGR Parameter ~ Arm Status + Time Point + Side of Lesion + Arm Status*Time Point + Side of Lesion*Time Point + Arm Status*Side of Lesion*Time Point + (1|Subject)’. For this equation, our fixed effects were arm status (ipsilesional versus contralesional arm), time point, and stroke-lesioned hemisphere. Fixed effects were assessed alone, and in combination with one another to examine interaction effects (asterisk between fixed effects in equation). We also included a random intercept effect for each participant (the “1|Subject” term in the equation) to reflect the differences in initial impairment severity each participant had. When looking at the model outputs (Supplementary Table 2), the “Intercept” term represents the model prediction of the contralesional arm score at the first time point for the parameter being assessed. All other values represent the difference in score from the prediction to the term in question (i.e., ipsilesional arm, time point, lesioned hemisphere). To verify whether the model predictions were accurate, we compared predicted values to actual scores for each parameter.

An example verification for the movement time parameter using values from Supplementary Table 2 is as follows: The model estimate for contralesional arm movement time z-score at the first time point was 5.09; the actual recorded mean contralesional arm movement time z-score at this time point was 4.6 ± 0.35. As the prediction was close to the actual value for the contralesional arm at the first time point, another time point was assessed and for the ipsilesional arm to determine if the model held accurate. To find the model prediction of the ipsilesional arm movement time z-score at the third time point, the following difference values were needed from the linear mixed model: ipsilesional arm at the first time point (-4.49), contralesional arm at the third time point (-3.12), and the interaction between the ipsilesional arm and the third time point (2.45). Adding these together with the intercept estimate (5.09) provided a final value of -0.07, which represented the movement time z-score for the ipsilesional arm at the third time point. The actual recorded mean ipsilesional arm movement time z-score at this time point was 0.01 ± 0.09. This verification was repeated for all parameters of the VGR task to determine that the linear mixed model equation was accurate for predicting z-scores.
