# Supplementary material for: Impairments of the ipsilesional upper-extremity in the first 6-months post-stroke
Source: J Neuroeng Rehabil. 2023 Aug 14;20:106. doi: 10.1186/s12984-023-01230-8 (PMC10424459; doi:10.1186/s12984-023-01230-8)
Supplement: Supplementary file 2 — Additional file 2. Table S1. Results of the ANOVA completed for the fixed effects of the linear mixed models for each of the 4 parameters of the VGR task. Bolded values indicate statistical significance at the 95% confidence level. FStat: F-statistic of the ANOVA test used for the linear mixed model. [file 12984_2023_1230_MOESM2_ESM.docx]

| Parameter of the VGR Task | Fixed Effects from Linear Mixed Models | | | | | | |
| --- | --- | --- | --- | --- | --- | --- | --- |
|  | Arm Status | Time Point | Arm Status with Time Point | Side of Lesion | Side of Lesion with Time Point | Side of Lesion with Arm Status | Side of Lesion with Arm Status and Time Point |
| Z-Task Score | **p=1.87x10^-24^**  **FStat=111** | **p=3.68x10^-22^**  **FStat=36.5** | p=7.16x10^-2^  FStat=2.34 | p=0.561  FStat=0.338 | p=0.738  FStat=0.421 | p=0.442  FStat=0.591 | p=0.720  FStat=0.447 |
| Reaction Time | **p=1.63x10^-8^**  **FStat=32.6** | **p=1.30x10^-11^**  **FStat=18.5** | p=0.368  FStat=1.05 | p=0.865  FStat=2.88x10^-2^ | p=0.146  FStat=1.80 | p=0.244  FStat=1.36 | p=0.468  FStat=0.847 |
| Initial Direction Error | **p=3.19x10^-31^**  **FStat=147** | **p=5.42x10^-21^**  **FStat=34.5** | **p=4.05x10^-5^**  **FStat=7.77** | p=0.253  FStat=1.31 | p=0.483  FStat=0.819 | p=0.368  FStat=0.813 | p=0.782  FStat=0.360 |
| Movement Time | **p=2.99x10^-38^**  **FStat=186** | **p=1.20x10^-25^**  **FStat=42.7** | **p=1.91x10^-9^**  **FStat=14.9** | p=8.92x10^-2^  FStat=2.90 | p=0.589  FStat=0.640 | p=8.84x10^-2^  FStat=2.91 | p=0.744  FStat=0.412 |

**Additional file 2: Table S1.** Results of the ANOVA completed for the fixed effects of the linear mixed models for each of the 4 parameters of the VGR task. Bolded values indicate statistical significance at the 95% confidence level. FStat: F-statistic of the ANOVA test used for the linear mixed model.
