# Supplementary material for: Impairments of the ipsilesional upper-extremity in the first 6-months post-stroke
Source: J Neuroeng Rehabil. 2023 Aug 14;20:106. doi: 10.1186/s12984-023-01230-8 (PMC10424459; doi:10.1186/s12984-023-01230-8)
Supplement: Supplementary file 3 — Additional file 3. Table S2. Estimates and corresponding p-values for all fixed effects of the linear mixed models for each parameter. Bolded values indicate statistical significance at the 95% confidence level. Bracketed values indicate the lower and upper bounds of each estimate, respectively. Ipsi: Ipsilesional Arm, LSL: Left-side lesion, TP: Time Point. Asterix indicates an interaction between fixed effects. [file 12984_2023_1230_MOESM3_ESM.docx]

**Additional file 3: Table S2.** Estimates and corresponding p-values for all fixed effects of the linear mixed models for each parameter. Bolded values indicate statistical significance at the 95% confidence level. Bracketed values indicate the lower and upper bounds of each estimate, respectively. Ipsi: Ipsilesional Arm, LSL: Left-side lesion, TP: Time Point. Asterix indicates an interaction between fixed effects

| Coefficient Name | Estimates and their Corresponding P-Values for Each Parameter | | | |
| --- | --- | --- | --- | --- |
|  | Z-Task Score | Reaction Time | Initial Direction Error | Movement Time |
| Intercept | **5.83 (5.18, 6.48)**  **p=1.23x10^-59^** | **2.04 (1.63, 2.44)**  **p=9.94x10^-22^** | **6.34 (5.61, 7.07)**  **p=1.70x10^-55^** | **5.09 (4.49, 5.69)**  **p=4.64x10^-54^** |
| Ipsi | **Δ-3.61 (-4.28, -2.94)**  **p=1.87x10^-24^** | **Δ-1.15 (-1.54, -0.75)**  **p=1.63x10^-8^** | **Δ-5.23 (-6.07, -4.38)**  **p=3.19x10^-31^** | **Δ-4.49 (-5.14, -3.85)**  **p=2.99x10^-38^** |
| LSL | Δ-0.295 (-1.29, 0.70)  p=0.561 | Δ-5.46x10^-2^ (-0.69, 0.58)  p=0.865 | Δ-0.666 (-1.81, 0.48)  p=0.253 | Δ-0.808 (-1.74, 0.12)  p=8.92x10^-2^ |
| TP2 | **Δ-2.17 (-2.85, -1.50)**  **p=3.77x10^-10^** | **Δ-0.868 (-1.26, -0.47)**  **p=1.82x10^-5^** | **Δ-2.53 (-3.38, -1.69)**  **p=6.18x10^-9^** | **Δ-2.46 (-3.11, -1.81)**  **p=2.35x10^-13^** |
| TP3 | **Δ-3.10 (-3.77, -2.43)**  **p=8.73x10^-19^** | **Δ-1.41 (-1.81, -1.02)**  **p=3.74x10^-12^** | **Δ-3.93 (-4.77, -3.08)**  **p=4.77x10^-19^** | **Δ-3.12 (-3.76, -2.47)**  **p=2.43x10^-20^** |
| TP4 | **Δ-3.14 (-3.81, -2.47)**  **p=2.94x10^-19^** | **Δ-1.15 (-1.54, -0.75)**  **p=1.44x10^-8^** | **Δ-3.68 (-4.53, -2.84)**  **p=4.86x10^-17^** | **Δ-3.34 (-3.99, -2.70)**  **p=6.09x10^-23^** |
| Ipsi*LSL | Δ-0.407 (-1.45, 0.63)  p=0.442 | Δ-0.365 (-0.98, 0.25)  p=0.244 | Δ0.606 (-0.71, 1.93)  p=0.368 | Δ0.877 (-0.13, 1.89)  p=8.84x10^-2^ |
| Ipsi*TP2 | Δ0.821 (-0.12, 1.76)  p=8.60x10^-2^ | Δ0.168 (-0.38, 0.72)  p=0.550 | **Δ1.59 (0.41, 2.77)**  **p=8.27x10^-3^** | **Δ2.06 (1.16, 2.97)**  **p=8.24x10^-6^** |
| Ipsi*TP3 | Δ1.05 (0.12, 1.99)  p=2.76x10^-2^ | Δ0.484 (-0.07, 1.03)  p=8.51x10^-2^ | **Δ2.65 (1.47, 3.83)**  **p=1.14x10^-5^** | **Δ2.45 (1.55, 3.35)**  **p=1.28x10^-7^** |
| Ipsi*TP4 | Δ1.14 (0.20, 2.07)  p=1.73x10^-2^ | Δ0.158 (-0.39, 0.71)  p=0.572 | **Δ2.36 (1.18, 3.54)**  **p=9.14x10^-5^** | **Δ2.82 (1.92, 3.72)**  **p=1.28x10^-9^** |
| LSL*TP2 | Δ-9.02x10^-3^ (-1.04, 1.03)  p=0.986 | Δ-0.649 (-1.26, -0.04)  p=3.79x10^-2^ | Δ0.345 (-0.97, 1.66)  p=0.606 | Δ0.595 (-0.41, 1.60)  p=0.245 |
| LSL*TP3 | Δ-1.98x10^-2^ (-1.06, 1.02)  p=0.970 | Δ-0.213 (-0.83, 0.40)  p=0.496 | Δ0.689 (-0.63, 2.00)  p=0.304 | Δ0.547 (-0.46, 1.55)  p=0.286 |
| LSL*TP4 | Δ-0.484 (-1.52, 0.55)  p=0.358 | Δ-0.525 (-1.14, 0.09)  p=9.21x10^-2^ | Δ-0.273 (-1.58, 1.04)  p=0.682 | Δ0.179 (-0.82, 1.18)  p=0.726 |
| LSL*Ipsi*TP2 | Δ0.171 (-1.28, 1.62)  p=0.817 | Δ0.551 (-0.30, 1.41)  p=0.206 | Δ-0.258 (-2.09, 1.57)  p=0.783 | Δ-0.784 (-2.19, 0.62)  p=0.272 |
| LSL*Ipsi*TP3 | Δ0.592 (-0.86, 2.05)  p=0.425 | Δ0.401 (-0.46, 1.26)  p=0.206 | Δ-0.639 (-2.48, 1.20)  p=0.495 | Δ-0.441 (-1.85, 0.96)  p=0.538 |
| LSL*Ipsi*TP4 | Δ0.741 (-0.71, 2.19)  p=0.316 | Δ0.646 (-0.21, 1.50)  p=0.138 | Δ0.280 (-1.55, 2.11)  p=0.764 | Δ-0.511 (-1.91, 0.89)  p=0.474 |
