# Supplementary material for: Impairments of the ipsilesional upper-extremity in the first 6-months post-stroke
Source: J Neuroeng Rehabil. 2023 Aug 14;20:106. doi: 10.1186/s12984-023-01230-8 (PMC10424459; doi:10.1186/s12984-023-01230-8)
Supplement: Supplementary file 4 — Additional file 4. Table S3. Significant changes in parameter scores from one time point to the next for all parameters presented. Bolded values indicate statistical significance of Chi-squared test at the 95% confidence level. Brackets: (Significant improvement in score with contralesional arm, significant degradation in score with contralesional arm, significant improvement in score with ipsilesional arm, significant degradation in score with ipsilesional arm). Chi: Chi-square test value. [file 12984_2023_1230_MOESM4_ESM.docx]

| Parameter of the VGR Task | Time Period Post-Stroke | | |
| --- | --- | --- | --- |
|  | 1-6 Weeks | 6-12 Weeks | 12-26 Weeks |
| Z-Task Score | **(41, 2 ,33, 5)**  **Chi=70.5** | **(17, 1 ,11, 2)**  **Chi=11.8** | (4, 3, 6, 7)  Chi=3.59 |
| Reaction Time | **(34, 6, 26, 1)**  **Chi=51.6** | **(11, 3, 5, 6)**  **Chi=7.01** | **(4, 12, 9, 4)**  **Chi=10.2** |
| Initial Direction Error | **(44, 2, 21, 0)**  **Chi=51.6** | **(30, 3, 6, 3)**  **Chi=22.4** | **(15, 9, 5, 4)**  **Chi=13.7** |
| Movement Time | **(50, 1, 14, 1)**  **Chi=50.3** | **(27, 2, 7, 3)**  **Chi=19.4** | **(16, 1, 7, 6)**  **Chi=11.0** |

**Additional file 4: Table S3.** Significant changes in parameter scores from one time point to the next for all parameters presented. Bolded values indicate statistical significance of Chi-squared test at the 95% confidence level. Brackets: (Significant improvement in score with contralesional arm, significant degradation in score with contralesional arm, significant improvement in score with ipsilesional arm, significant degradation in score with ipsilesional arm). Chi: Chi-square test value.
