# Supplementary material for: Impairments of the ipsilesional upper-extremity in the first 6-months post-stroke
Source: J Neuroeng Rehabil. 2023 Aug 14;20:106. doi: 10.1186/s12984-023-01230-8 (PMC10424459; doi:10.1186/s12984-023-01230-8)
Supplement: Supplementary file 5 — Additional file 5. Table S4. Results of the linear regressions comparing the effect of left-versus-right side lesions on impairment severity for the ipsilesional and contralesional arm for all parameters presented. Bolded values indicate statistical significance at the 95% confidence level. LSL: Left-side lesion, RSL: Right-side lesion, KS: Kolmogorov-Smirnov test value. [file 12984_2023_1230_MOESM5_ESM.docx]

| Parameter of the VGR Task | Statistical Parameter | | |
| --- | --- | --- | --- |
|  | Linear Regression P-Value | Linear Regression Coefficient of Determination | Kolmogorov-Smirnov Test and Resulting P-Value |
| Z-Task Score | LSL: **p = 4.86 x 10^-2^**  RSL: **p = 4.67 x 10^-5^** | LSL: R^2^ = 0.089  RSL: R^2^ = 0.247 | KS = 0.243  p = 0.618 |
| Reaction Time | LSL: **p = 2.77 x 10^-7^**  RSL: **p = 6.06 x 10^-10^** | LSL: R^2^ = 0.470  RSL: R^2^ = 0.480 | KS = 0.259  p = 0.472 |
| Initial Direction Error | LSL: p = 0.836  RSL: p = 0.066 | LSL: R^2^ = 1.03 x 10^-3^  RSL: R^2^ = 5.63 x 10^-2^ | KS = 0.196  p = 1.00 |
| Movement Time | LSL: **p = 5.98 x 10^-5^**  RSL: **p = 1.46 x 10^-4^** | LSL: R^2^ = 0.322  RSL: R^2^ = 0.218 | KS = 0.262  p = 0.450 |

**Additional file 5: Table S4.** Results of the linear regressions comparing the effect of left-versus-right side lesions on impairment severity for the ipsilesional and contralesional arm for all parameters presented. Bolded values indicate statistical significance at the 95% confidence level. LSL: Left-side lesion, RSL: Right-side lesion, KS: Kolmogorov-Smirnov test value.
