# Supplementary material for: Impairments of the ipsilesional upper-extremity in the first 6-months post-stroke
Source: J Neuroeng Rehabil. 2023 Aug 14;20:106. doi: 10.1186/s12984-023-01230-8 (PMC10424459; doi:10.1186/s12984-023-01230-8)
Supplement: Supplementary file 6 — Additional file 6. Table S5. Results of the ANOVA completed for the fixed effects of the linear mixed models for each of the 4 parameters of the VGR task with neglect accounted for. Bolded values indicate statistical significance at the 95% confidence level. FStat: F-statistic of the ANOVA test used for the linear mixed model. [file 12984_2023_1230_MOESM6_ESM.docx]

|  | Time Point | | | |
| --- | --- | --- | --- | --- |
| Number of Participants | Neglect | Arm Status with Neglect | Time Point with Neglect | Arm Status with Time Point and Neglect |
| Z-Task Score | p=0.350  FStat=0.87 | p=0.559  FStat=0.34 | p=0.670  FStat=0.52 | p=0.413  FStat=0.96 |
| Reaction Time | p=0.040  FStat=4.23 | p=0.066  FStat=3.39 | p=0.189  FStat=1.60 | p=0.299  FStat=1.23 |
| Initial Direction Error | p=0.162  FStat=1.96 | p=0.322  FStat=0.98 | p=0.239  FStat=1.41 | p=0.366  FStat=1.06 |
| Movement Time | p=0.394  FStat=0.73 | p=0.374  FStat=0.79 | p=0.645  FStat=0.55 | p=0.677  FStat=0.51 |

**Additional file 6: Table S5.** Results of the ANOVA completed for the fixed effects of the linear mixed models for each of the 4 parameters of the VGR task with neglect accounted for. Bolded values indicate statistical significance at the 95% confidence level. FStat: F-statistic of the ANOVA test used for the linear mixed model.
