# Supplementary material for: Impairments of the ipsilesional upper-extremity in the first 6-months post-stroke
Source: J Neuroeng Rehabil. 2023 Aug 14;20:106. doi: 10.1186/s12984-023-01230-8 (PMC10424459; doi:10.1186/s12984-023-01230-8)
Supplement: Supplementary file 7 — Additional file 7. Table S6. Summary of participant scores on the Modified Ashworth Scale for the contralesional arm at each of the four time points. Bracketed numbers represent the number of participants at each score at each time point. Modified Ashworth Scale scores follow the following order in brackets: (0, 1, 1+, 2, 3, 4). Modified Ashworth Scale was not collected for 13 participants at 1-week post-stroke, 7 participants at 6-weeks post-stroke, 9 participants at 12-weeks post-stroke, and 3 participants at 26-weeks post-stroke. [file 12984_2023_1230_MOESM7_ESM.docx]

|  | Time Point | | | |
| --- | --- | --- | --- | --- |
|  | 1-Week Post-Stroke | 6-Weeks Post-Stroke | 12-Weeks Post-Stroke | 26-Weeks Post-Stroke |
| Number of Participants | (70, 11, 7, 4, 1, 0) | (67, 18, 7, 5, 2, 0) | (65, 12, 9, 8, 3, 0) | (74, 9, 2, 12, 6, 0) |

**Additional file 7: Table S6.** Summary of participant scores on the Modified Ashworth Scale for the contralesional arm at each of the four time points. Bracketed numbers represent the number of participants at each score at each time point. Modified Ashworth Scale scores follow the following order in brackets: (0, 1, 1+, 2, 3, 4).

Modified Ashworth Scale was not collected for 13 participants at 1-week post-stroke, 7 participants at 6-weeks post-stroke, 9 participants at 12-weeks post-stroke, and 3 participants at 26-weeks post-stroke.
